# Is Midline Uterosacral Plication Anterior Colporrhaphy Combo (MUSPACC) Procedure a Good Option in Management of Vaginal Vault Prolapse and Cystocele?

**DOI:** 10.3390/medicina62040709

**Published:** 2026-04-08

**Authors:** Aiste Ugianskiene, Caroline Sollberger Juhl, Karin Glavind

**Affiliations:** Gynecological-Obstetrical Department, Aalborg University Hospital, Reberbansgade 15, 9000 Aalborg, Denmark; c.juhl@rn.dk (C.S.J.); kagl@rn.dk (K.G.)

**Keywords:** complications, cystocele, MUSPACC, patient satisfaction, urinary incontinence, vaginal vault prolapse

## Abstract

*Background and Objectives*: After the publication by Haylen et al. introducing the MUSPACC (midline uterosacral plication anterior colporrhaphy combination) procedure, we implemented this technique in our clinical practice for the treatment of cystocele and vaginal vault prolapse (VVP). The aims of this study were to evaluate peri- and postoperative complications, as well as vaginal and urinary symptoms, including patient satisfaction 3 months postoperatively. *Materials and Methods*: A retrospective analysis was conducted on 58 women who underwent MUSPACC over a five-year period. Patient-reported outcomes were assessed using three prolapse-related items from the International Consultation on Incontinence–Vaginal Symptoms (ICIQ-VS) and the International Consultation on Incontinence Questionnaire–Urinary Incontinence Short Form (ICIQ-UI SF), administered preoperatively and at three months following surgery. Demographic characteristics, as well as perioperative and postoperative complications, were obtained through review of medical records. *Results*: At follow-up, patients demonstrated improvement in vaginal symptoms, with the mean preoperative ICIQ-VS score decreasing from 15.2 to 1.16. Among those with preoperative urinary incontinence (UI), 42.1% became completely dry after MUSPACC procedure alone. Three patients (15%) developed de novo UI. Perioperative complications occurred in one patient. The postoperative complication rate was 20.7% (12/58), including one patient who experienced a postoperative fistula between the right ureter and vagina. No further surgeries were required. Overall, 96.4% of patients were satisfied postoperatively. *Conclusions*: MUSPACC procedure appears to be an effective treatment for VVP and cystocele, with improvement in vaginal and urinary symptoms, high patient satisfaction, and a low rate of serious complications. Routine perioperative cystoscopy is now performed for immediate detection and management of urinary tract injuries.

## 1. Introduction

The incidence of vaginal vault prolapse (VVP) has been reported to be 11.6% among women undergoing hysterectomy for prolapse, compared with 1.8% in those undergoing hysterectomy for other benign conditions [1]. The proportion of patients requiring surgical correction for VVP is estimated to range between 6% and 8% [2].

The surgical management of VVP has been extensively studied over several decades, with a variety of approaches described, including vaginal, abdominal, and laparoscopic techniques.

However, approaches vary depending on the surgeon’s experience and institutional practices.

Patients with VVP often present with concurrent pelvic floor defects, e.g., a cystocele, rectocele or enterocele [3].

After reading the publication by Haylen BT et al. [4] we changed our clinical practice and adopted the MUSPACC procedure. The procedure consists of an extraperitoneal midline uterosacral plication above the vaginal vault combined with anterior colporrhaphy performed through a single midline anterior vaginal wall incision.

The initial report by Haylen et al. demonstrated that MUSPACC is a safe procedure with minimal blood loss and a low risk of the ureteric injury [4]. In addition, short-term anatomical outcomes were encouraging.

The aims of this study were to evaluate peri- and postoperative complications, as well as vaginal and urinary symptoms, including patient satisfaction at 3 months postoperatively.

## 2. Materials and Methods

This retrospective cohort study included all women who underwent the MUSPACC procedure at our department over a five-year period from January 2019 to January 2024. A total of 58 patients were identified.

All patients completed three modified prolapse-related questions from the International Consultation on Incontinence-Vaginal Symptoms (ICIQ-VS) [5], along with the International Consultation on Incontinence—Urinary Incontinence Short Form (ICIQ-UI SF) [6,7], both preoperatively and at three months postoperatively. The three modified ICIQ-VS questions assessed the presence and severity of bulge symptoms:

“Do you feel a lump or bulge coming out of your vagina, or can you feel a lump in or outside your vagina?” (never (0), occasionally (1), sometimes (2), most of the time (3) or all of the time (4));

“How much does this bother you?” (not at all (0), a little (1), some (2), very much (3));

“How much does this affect your daily life?” (0–10).

A composite score was calculated, ranging from 0 (asymptomatic) to 17 (maximum symptom burden).

The ICIQ-UI-SF was used to evaluate the severity of urinary incontinence and its impact on quality of life. The questionnaire consists of three scored items and one unscored self-diagnostic item. The total score ranges from 0 (continent) to 21 (severe incontinence). The unscored item was used to classify the type of UI as stress, urge, mixed, or undefined. Undefined UI was defined as leakage after voiding or leakage without obvious reason. De novo UI was defined as an ICIQ-UI-SF score > 0 at 3 months postoperatively. The ICIQ-UI SF is a validated questionnaire in English and has been translated into Danish.

Demographic variables included age, body mass index (BMI), parity, history of cesarean section, and previous prolapse and incontinence surgery. All patients underwent a standardized preoperative physical examination.

The MUSPACC procedure was performed according to Haylen et al. [4] combining local anesthesia with general anesthesia. A midline anterior vaginal wall incision was made from the bladder neck to the vaginal vault. The bladder and pubocervical fascia were mobilized using both blunt and sharp dissection, laterally from the vaginal epithelium and dorsosuperiorly from the extraperitoneal aspect of the vaginal vault. The intermediate portion of the uterosacral ligament (USL) was identified dorsolaterally and plicated in the midline above the vaginal cuff.

Midline plication of the contralateral USLs was performed using a 2-0 delayed absorbable monofilament suture. A standard anterior colporrhaphy with 2-0 delayed continuous absorbable polyfilament suture in one row was performed. The last stitch involved the conjoined sacrouterine ligaments. The vaginal epithelium was then trimmed, and the anterior vaginal wall was closed with a continuous 2-0 absorbable suture. The urethral Foley catheter was removed at the end of the surgery.

All procedures were performed by the four surgeons using a standardized technique.

Follow-up was conducted at three months postoperatively, either clinically or via telephone, in 56 patients. Data were missing for two patients.

All data extracted were entered into The Research Electronic Data Capture (RedCap) system, which is a browser-based platform for clinical research data management.

The present study was designed as a descriptive cohort analysis focused on feasibility, safety, and short-term outcomes of MUSPACC.

### Statistical Analysis

Descriptive statistics were used to summarize the study population.

The study was approved and registered at The Health department, Region Nord Jylland, Denmark, ID number K2024-175. The study was a quality assurance project; therefore, there was no need to obtain informed consent for each patient.

## 3. Results

A total of 58 patients underwent MUSPACC procedure during the study period. No concomitant procedures were performed.

Demographic characteristics and the preoperative stages of prolapse are shown in Table 1.

A total of 56 patients attended the 3-month follow-up. At the follow-up, patients demonstrated improvement in vaginal symptoms, with the mean ICIQ-VS score decreasing from 15.2 preoperatively to 1.16 postoperatively.

**Figure 1:**
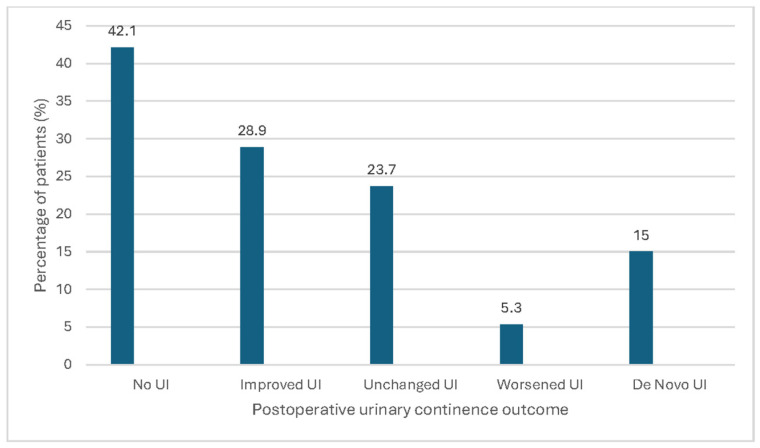
Changes in urinary continence status 3 months after MUSPACC surgery.

Postoperative changes in urinary incontinence are shown in Figure 1.

Sixteen patients (42.1%) with preoperative UI became completely continent following MUSPACC procedure. Three patients (15%) developed de novo UI 3 months after surgery. A total of 11 patients (55%), who remained incontinent after prolapse surgery according to the ICIQ-UI SF, showed improvement in UI, while two patients (10%) reported worsening symptoms. The mean ISIQ-UI-SF score before surgery was 6.72, which declined to 3.75 postoperatively.

Regarding patient satisfaction, 54 patients (96.4%) felt “cured”, “greatly improved” and “somewhat improved”, while two patients reported no improvement at 3 months follow-up.

Perioperative complications occurred in one patient: a small bladder perforation, which occurred during dissection and was recognized and repaired at the time of the operation. Twelve patients (20.7%) experienced different postoperative complications: postoperative urinary tract infection (*n* = 5), postoperative voiding dysfunction (*n* = 4), postoperative bleeding/hematoma (*n* = 3), postoperative pain (*n* = 2), and postoperative infection (*n* = 1). All complications were managed conservatively without reoperation.

One patient developed a postoperative ureterovaginal fistula. The patient was a 74-year-old woman, with a history of vaginal hysterectomy, presenting with a grade 3 cystocele and grade 2 apical prolapse. A MUSPACC operation was completed without perioperative complications. and the patient was discharged the following day. One month postoperatively, she presented with continous leakage of clear fluid per vagina without signs of infection or pain. Vaginal examination revealed leakage of clear fluid from the vaginal cicatrice. Ultrasound showed a normal bladder capacity but right-sided hydronephrosis. The vaginal fluid demonstrated elevated creatinine levels (>3500 umol/L) diagnostic of urine leakage. To facilitate detection of the vesicovaginal fistula, a sterile solution of methylene blue (MB) diluted in saline was instilled into the bladder; however, no leakage was observed. Computed tomography (CT)-urography demonstrated right-sided hydroureter and hydronephrosis, with contrast visible in the vagina, indicating a urinary tract injury. A nephrostomy catheter was placed, and symptoms of urinary incontinence diminished. In addition, an antegrade pyelography further revealed delayed passage of contrast through the distal right ureter, along with leakage through the vaginal wall, confirming the presence of a fistula. The patient was referred to a specialized center for consideration of robot-assisted laparoscopy with neo-implantation of the right ureter. Meanwhile, a right double-J (JJ) ureteral stent was inserted. At six weeks follow-up, the stent was removed after normal findings on CT urography. Renography confirmed restoration of normal renal function, thereby eliminating the need for ureteral reimplantation

## 4. Discussion

This study demonstrated an improvement in vaginal and urinary symptoms and a high patient satisfaction. The rate of perioperative and serious postoperative complications was low.

Vaginal symptom improvement at 3 months follow-up was notable, although direct comparison with other studies is limited due to lack of published short-term data. Haylen et al. [4] described subjective and objective results after MUSPACC procedure (*n* = 41) at early follow-up (mean 6.5 weeks; range 4–9 weeks). All patients were free of prolapse symptoms and no recurrence of apical prolapse was observed.

In our cohort, 65.5% of patients had concomitant UI preoperatively. Among these, 42.1% became completely continent following the MUSPACC procedures alone, and additionally almost 1/3 of women experienced improvement in UI 3 months after surgery. These findings support a staged approach, avoiding routine concomitant anti-incontinence surgery and allowing time to assess the effect of prolapse repair alone [8]. Three patients (15%) developed de novo UI 3 months after MUSPACC procedure, consistent with previous reports following vaginal prolapse surgery [9,10,11,12]. No data on MUSPACC procedure and risk of de novo UI has been found. Khayyami et al. [10] included 1198 urinary continent women who underwent POP surgery. The risk of de novo UI was 15%. Their study also showed that twice as many women with BMI ≥ 30 had de novo UI compared with women with BMI < 25 [10]. We could not confirm their findings, as all women with de novo UI, in our study, had BMI < 25.

Preoperatively, 63.8% of our patients had stage 3 cystocele and 58.6% stage 1 vaginal vault prolapse (Table 1). In Haylen et al.’s [4] paper, patients who underwent MUSPACC procedure preoperatively had anterior vaginal prolapse mean stage 2.0 and apical vaginal prolapse mean stage 1.4. Nearly 42% of patients had stage 2 and stage 3 vaginal vault prolapse preoperatively, suggesting the MUSPACC procedure may be suitable for more advanced vaginal vault prolapse.

The most common postoperative complication was urinary tract infection (UTI), which occurred in 9% of patients. Patients with symptoms of urinary tract infection, in our country, usually contact their general practitioner and receive treatment. No patients needed hospitalization due to UTI.

Haylen BT et al. [4] reported no ureteric injuries in their initial series of 41 patients or in over 300 MUSPACC procedures performed before and after publication. In contrast to this paper, the most serious complication after MUSPACC procedure, in our study, was a postoperative fistula between the right ureter and vagina.

An advantage of MUSPACC over alternative vaginal approaches is that it is a minimally invasive procedure that can be performed in a day-care setting. However, pelvic organ surgeons should remember that serious urinary tract injuries, although rare, can occur.

Prior to this case, cystoscopy was not a standard during MUSPACC procedures. Routine cystoscopy performed perioperatively may have facilitated earlier detection and management of this complication. Previous studies by Gilmour et al. and Sahai et al. [13,14] support the use of cystoscopy to improve detection of urinary tract injuries during pelvic floor surgery.

Routine cystoscopy has now been introduced, and no peri- or postoperative urinary tract injuries have been experienced since this alteration in procedure.

Our study revealed that 96.4% of patients reported high satisfaction, and no worsening of prolapse symptoms was noted postoperatively. Two patients stated no improvement at 3 months follow-up; both patients had experienced postoperative complications (postoperative voiding dysfunction, UTI, hematoma and pain) and persistent urinary symptoms, highlighting the multifactorial nature of the postoperative satisfaction.

The strength of this study is that the same questionnaire was used both pre- and postoperatively, allowing for accurate assessment of symptom changes. Additionally, data were recorded prospectively at the time of clinical event, improving reliability.

Limitations include the small cohort size and short-term (3 months) follow-up period; consequently, the recurrence rate may increase further with longer follow-up. No patients were tested postoperatively for UI, but patients who scored 0 on ISIQ-UI SF were considered continent. Postoperative complications were not formally graded using a standardized classification system such as Clavien–Dindo.

Additional studies are required to validate our results.

## 5. Conclusions

The MUSPACC procedure appears to be an effective surgical option for the treatment of vaginal vault prolapse and cystocele. It is associated with improvement in vaginal and urinary symptoms, high patient satisfaction and a low rate of serious postoperative complications. Routine perioperative cystoscopy is now performed for immediate detection and management of urinary tract injuries.

## Figures and Tables

**Table 1:** Demographics of the study population.

Characteristic	Value
Age, years	
Mean	68
Median (range)	71 (48–87)
Parity, median (range)	2 (1–5)
Body mass index (BMI), kg/m^2^, mean (range)	26.8 (20.1–36.1)
Smoking, n (%)	
Yes	5 (8.6)
No	53 (91.4)
Alcohol overuse, n (%)	
Yes	1 (1.7)
No (as less than 10 items pr. week)	57 (98.3)
Hypertension, n (%)	
Yes	29 (50.0)
No	29 (50.0)
Diabetes, n (%)	
Yes	8 (13.8)
No	50 (86.2)
Previous cesarean section, n (%)	1 (1.7)
Previous hysterectomy for prolapse, n (%)	15 (25.9)
Previous prolapse surgery, n (%)	35 (60.3)
Previous anti-incontinence surgery, n (%)	1 (1.7)
Anterior vaginal wall prolapse stage, n (%)	
Stage I	0
Stage II	21 (36.2)
Stage III	37 (63.8)
Stage IV	0
Anterior wall prolapse stage, mean (range)	2.6 (2–3)
Vaginal vault prolapse stage, n (%)	
Stage I	34 (58.6)
Stage II	18 (31.0)
Stage III	6 (10.4)
Stage IV	0
Vaginal vault prolapse stage, mean (range)	1.5 (1–3)

No alcohol defined as less than 10 items.

## Data Availability

The original contributions presented in this study are included in the article. Further inquiries can be directed to the corresponding author.

## Abbreviations

The following abbreviations are used in this manuscript:

VVP	vaginal vault prolapse
MUSPACC	midline uterosacral plication anterior colporrhaphy combo
ICIQ-VS	International Consultation on Incontinence—Vaginal Symptoms
ICIQ-UI SF	International Consultation on Incontinence—Urinary Incontinence Short Form
UI	urinary incontinence
UTI	urinary tract infection
